# Molecular Imaging for Comparison of Different Growth Factors on Bone Marrow-Derived Mesenchymal Stromal Cells' Survival and Proliferation *In Vivo*

**DOI:** 10.1155/2016/1363902

**Published:** 2016-06-21

**Authors:** Hongyu Qiao, Ran Zhang, Lina Gao, Yanjie Guo, Jinda Wang, Rongqing Zhang, Xiujuan Li, Congye Li, Yundai Chen, Feng Cao

**Affiliations:** ^1^Department of Cardiology, Xijing Hospital, Fourth Military Medical University, Xi'an 710032, China; ^2^Department of Cardiology, Chinese PLA General Hospital, Beijing 100853, China

## Abstract

*Introduction*. Bone marrow-derived mesenchymal stromal cells (BMSCs) have emerged as promising cell candidates but with poor survival after transplantation. This study was designed to investigate the efficacy of VEGF, bFGF, and IGF-1 on BMSCs' viability and proliferation both* in vivo* and* in vitro* using bioluminescence imaging (BLI).* Methods*. BMSCs were isolated from *β*-actin-Fluc^+^ transgenic FVB mice, which constitutively express firefly luciferase. Apoptosis was induced by hypoxia preconditioning for up to 24 h followed by flow cytometry and TUNEL assay. 10^6^ BMSCs with/without growth factors were injected subcutaneously into wild type FVB mice's backs. Survival of BMSCs was longitudinally monitored using bioluminescence imaging (BLI) for 5 weeks. Protein expression of Akt, p-Akt, PARP, and caspase-3 was detected by Western blot.* Results*. Hypoxia-induced apoptosis was significantly attenuated by bFGF and IGF-1 compared with VEGF and control group* in vitro* (*P* < 0.05). When combined with matrigel, IGF-1 showed the most beneficial effects in protecting BMSCs from apoptosis* in vivo.* The phosphorylation of Akt had a higher ratio in the cells from IGF-1 group.* Conclusion*. IGF-1 could protect BMSCs from hypoxia-induced apoptosis through activation of p-Akt/Akt pathway.

## 1. Introduction 

Heart failure (HF) is a severe clinical syndrome in patients with cardiovascular diseases that leads to frequent hospitalization, poor quality of life, and shortened life expectancy [1]. The pathogenesis of this syndrome is traditionally thought to result from structural and functional abnormalities in the myocardium [2]. Therefore, critical roles for cardiomyocyte's death in HF have received much attention in recent years [2, 3]. For the patients with refractory end-stage HF, although heart transplantation has been considered as a standard therapy [1], it is still limited in clinic due to shortness of heart donors.

Cardiac regenerative medicine and stem cell therapy may be promising therapeutic strategies for HF [4]. Several candidates of stem cells such as embryonic stem cells (ESCs) [5], fetal stem cells (FSCs) [6], mesenchymal stem cells (MSCs) [7–9], skeletal myoblasts [10], resident cardiac stem cells [11–13], and induced pluripotent stem cells (iPSCs) [14] have been widely investigated in the recent decade, some of which have got encouraging results. The bone marrow-derived mesenchymal stromal cells (BMSCs) are specifically valuable for their multipotent, immune-privileged [15, 16], and easily expanded* ex vivo* behaviors [17]. Ongoing clinical trials are investigating the potential benefit of cardiac progenitor cells and BMSCs [18]. However, the available evidence implicated that the reactions of host environment always impaired cell survival and biological functions [19–21]. Therefore, optimizing the fate of transplanted stem cells deserves a high priority in both basic and clinical translational medicine.

Applications of growth factors may be one of the promising modification methods, since vascular endothelial growth factor (VEGF), insulin growth factor-1 (IGF-I), and basic fibroblast growth factor (bFGF) have been proved to have pivotal functions upon stem cells' proliferation, differentiation, and many other biological processes [22–25]. Meanwhile, growth factors produce regulatory effects on heart failure [26, 27]. Matrigel as 3D skeletonization has been developed to create a bioactive environment with multiple growth factors [28] and the presence of matrigel was a premise for cardiomyocytes to extend cell-cell contact in collagen matrix [29]. Converging what was mentioned above, the present work was designed to investigate the effects of VEGF, bFGF, and IGF-1 on mouse BMSCs* via* molecular imaging and to clarify the molecular mechanism of these phenomena.

## 2. Material and Methods

### 2.1. Animals

*β*-actin-Fluc^+^ (firefly luciferase) transgenic mice were raised on a FVB background to constitutively express firefly luciferase driven by *β*-actin promoter in all tissues and organs, including bone marrow cell populations. The procedures of mice were in accordance with the Guiding Principles for the Care and Use of Animals by the Fourth Military Medical University and met the Chinese guidelines for experimental animals. The protocol was approved by the Ethics Review Board of the Fourth Military Medical University.

### 2.2. Isolation and Cultivation of BMSCs

*β*-actin-Fluc^+^ transgenic mice were sacrificed by euthanasia and immersed in 75% alcohol for 10 min. BMSCs were harvested from the long bones and centrifuged at 1000 ×g for 10 min. The supernatant was abandoned and then the cellular pellets were resuspended with complete medium (80% DMEM/F12, 15% fetal bovine serum, 100 U/L penicillin, 0.1 g/L streptomycin, 1 mL glutamine, and some 20% NaHCO_3_). The cells were incubated in 25 cm^2^ culture bottles with the complete medium. The bottles were maintained in a humidified environment containing 5% CO_2_ and 90% humidity at 37°C. The medium was refreshed every 3 days. When cells reached 80% confluence, they were propagated at the rate of 1 : 2. We chose the third passage for experiment and inverted phase contrast microscope was used to identify the morphology of cells.

### 2.3. Characteristics of BMSCs by Flow Cytometry

The third-passage cells were harvested and adjusted to the concentration at 1 × 10^6^/mL, which were then incubated in flow cytometry buffer (FCB: 2% FBS and 0.2% Tween-20 in PBS) containing fluorescein phycoerythrin-conjugated monoclonal rabbit anti-mouse CD34, CD44, CD45, and CD90 (BD Pharmingen, USA) for 30 min at 4°C. PBS was added and the suspensions were centrifuged at 1500 ×g for 5 min. The pellets were resuspended in PBS. Cells were analyzed by FACScan cytometer (BD Pharmingen) according to the manufacturer's protocol.

### 2.4. *In Vitro* Reporter Gene Imaging and Assays

The third-passage BMSCs at different concentrations were seeded in 24-well plates, respectively, and washed thoroughly in PBS after 24 h. D-Luciferin (10 *μ*g/mL) was then added and incubated for 1 min. The charge-coupled device (CCD, dual-modality) camera with Xenogen* In Vivo* Imaging System (IVIS, Caliper Life Sciences, USA; binning: 4; F/Stop: 1; exposure time: 1 min) and Living Imaging Version 4.0 software was used to analyze BLI signals. Peak intensity of BLI signals was expressed in average radiance (photons/second/cm^2^/steradian, P·s^−1^·cm^−2^·sr^−1^) from a fixed-area region of interest (ROI).

### 2.5. Flow Cytometry Analysis of Cell Apoptosis

Cells (1 × 10^6^/well) were seeded in 6-well plates. 24 h later, VEGF, bFGF, and IGF-1 (Peprotech, USA) were added to the marked plates, respectively, at the concentration of 20 ng/mL. The plates were exposed to hypoxia (0.5% O_2_, 5% CO_2_) for another 24 h. Apoptosis was determined by detecting phosphatidylserine exposure on cell plasma membrane with the fluorescent dye Annexin-V-FITC Apoptosis Detection Kit (BD Pharmingen, USA) according to the manufacturer's instructions. Briefly, BMSCs incubated with different growth factors were harvested, washed with PBS, and stained with Propidium Iodine and Annexin-V-FITC. The presence of Annexin-V-FITC-positive cells excluding PI at early time intervals suggested hypoxia-induced cell death by apoptosis. The data was analyzed by flow cytometry (BD FACSAria, USA) with CellQuest research software.

### 2.6. Assessment of Apoptosis by TUNEL Staining

Hypoxia-treated cells were preconditioned with VEGF, IGF-1, and bFGF and then collected for terminal deoxynucleotidyl transferase-mediated dUTP nick end labeling (TUNEL) apoptosis assay using a Cell Death Detection Kit (Promega, Madison, WI, USA) according to the manufacturer's instructions. DAPI staining was performed for total nuclei quantification. The results were expressed as the proportion of the TUNEL-positive BMSCs to the total BMSCs.

### 2.7. Bioluminescence Imaging of Engrafted BMSCs* In Vivo*

BMSCs were isolated from 6-week *β*-actin-Fluc^+^ transgenic mice (15–20 g). Eight-week-old inbred mice were applied as host mice. The third-passage BMSCs were harvested and adjusted to 10^6^ in each group. We administered the mixture of 10^6^ BMSCs, 20 *μ*L melt matrigel, and 1 *μ*L growth factor (20 ng/*μ*L) subcutaneously through a 27 g needle into the host mice's backs. The drug administration regions were BMSCs and matrigel at top left; BMSCs, matrigel, and VEGF at top right; BMSCs, matrigel, and bFGF at bottom left; BMSCs, matrigel, and IGF-1 at bottom right. Similar treatments were performed in another five mice. The biological signals were tracked* via* Xenogen* In Vivo* Imaging System (IVIS, Caliper Life Sciences, USA) at 0 d, 1 d, 3 d, 5 d, 7 d, 14 d, 21 d, 28 d, and 35 d. The survival of BMSCs* in vivo* was evaluated by the number of photons detected at predetermined time after administration.

### 2.8. Determination of BMSCs' Viability* In Vivo*

After transplantation for 4 weeks, mice were sacrificed to excise the masses for frozen sections. Biotin labeling fluid (50 *μ*L) (TdT enzyme (2 *μ*L) and Biotin-dUTP (48 *μ*L)) and glycine (0.2 mL) as a labeling stopper were sequentially added to the samples. They were incubated with Streptavidin-HRP for 10 min and diaminobenzidine (0.5 mL) for 25 min at room temperature. Nuclei were stained with hematoxylin (R&D Systems, USA). Finally, the samples were imaged using laser confocal microscope (FluoView-FV1000, Olympus, Japan).

### 2.9. Western Blot Analysis

For* in vitro* part, cells preconditioned with VEGF, IGF-1, and bFGF and cultured under hypoxia for 24 h were harvested. For* in vivo* part, 3 weeks after injecting the mixture of BMSCs, matrigel, and growth factors in mice's backs, masses were taken out and protein lysis was harvested. Protein concentrations were determined with a bicinchoninic acid (BCA) assay kit (Pierce, Rockford, IL). The proteins were separated by 10% SDS-PAGE gels and transferred onto nitrocellulose membrane. Membranes were blocked with Tris buffered saline-Tween-20 containing 5% nonfat milk and incubated with primary antibodies (Cell Signaling Technology, USA) (dilution 1 : 2000 for anti-Akt and anti-*β*-actin; 1 : 1000 for anti-pho-Akt, anti-caspase-3, and anti-PARP) overnight at 4°C. The membranes were incubated with secondary antibodies (Abcam, Cambridge, MA, USA) conjugated with horseradish peroxidase for 1 h. The target protein was detected by enhanced chemiluminescence system (Amersham Bioscience, Fairfield, Connecticut, USA) and quantified by Quantity One Analysis Software (version 4.5, Bio-Rad, USA).

### 2.10. Statistical Analysis

Results were expressed as mean ± SD. Prism 5.0 (GraphPad Software Inc., San Diego, CA, USA) was used to make statistical analysis. Statistical comparisons for different groups were performed using one-way or two-way analysis of variance (ANOVA). *P* values < 0.05 were considered statistically significant.

## 3. Results

### 3.1. Characterization of BMSCs

**Figure 1:**
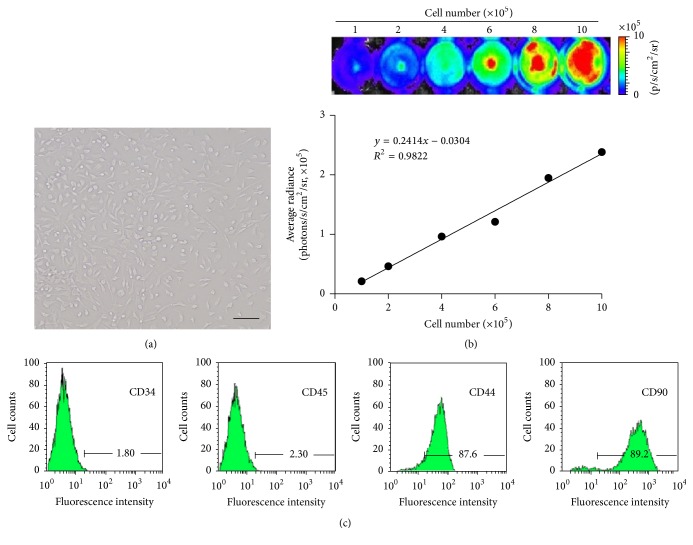
Characterization of bone marrow-derived mesenchymal stromal cells (BMSCs). (a) Morphology of BMSCs at 5 d; after cell culture for 24 h, only several adherent cells were found. Five to seven days later, a colony made of hundreds of cells was recognized and mononuclear cell's shapes were fusiformis or polygon. Magnification: ×100. (b) Optical imaging of *β*-actin-Fluc^+^ BMSCs and the linear correlation between cell number and densities of Fluc signals; the densities of Fluc signals were strongly correlated to the number of cells (*R* ^2^ = 0.95, *P* < 0.01). (c) Flow cytometry analysis of mouse BMSCs labeled with phycoerythrin/allophycocyanin-conjugated CD34, CD45, CD44, and CD90; most BMSCs expressed typical stem cell markers CD90 (89.2%) and CD44 (87.6%) and low levels of hematopoietic markers CD45 (2.3%) and CD34 (1.8%). Bar scale = 100 *μ*m; *n* = 6.

After cell culture for 24 h, only several adherent cells were found. Five to seven days later, a colony made of hundreds of cells was recognized and mononuclear cell's shapes were fusiformis or polygon (Figure 1(a)). The densities of Fluc signals were strongly correlated to the number of cells (*R* ^2^ = 0.95, *P* < 0.01) (Figure 1(b)). Most BMSCs expressed typical stem cell markers CD90 (89.2%) and CD44 (87.6%) and low levels of hematopoietic markers CD45 (2.3%) and CD34 (1.8%) (Figure 1(c), *n* = 6 in each group).

### 3.2. Growth Factors Decreased the Apoptosis of BMSCs

**Figure 2:**
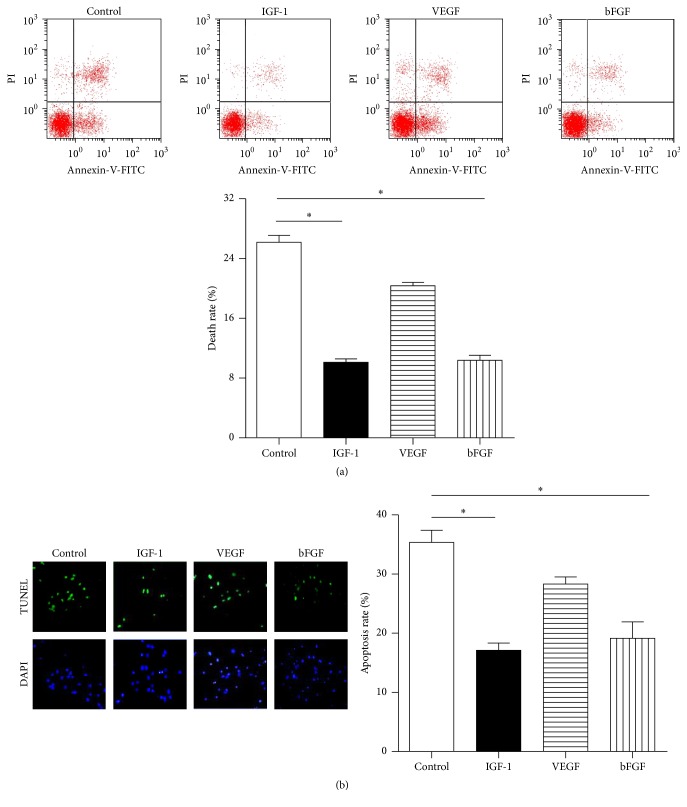
The effects of pretreatment with VEGF, IGF-1, and bFGF on hypoxia-induced apoptosis of bone marrow-derived mesenchymal stromal cells (BMSCs). (a) Apoptosis analysis of BMSCs by flow cytometry; when exposed to hypoxia, BMSCs treated with IGF-1 and bFGF underwent less apoptosis compared with control (10.2 ± 1.4% and 10.7 ± 2.5% versus 26.1 ± 3.4%; *P* < 0.05; *n* = 6 in each group), while the apoptosis rate of VEGF was 20.7 ± 1.9%. (b) Apoptosis analysis of BMSCs by TUNEL assay; BMSCs with bFGF and IGF-1 showed a lower calculated apoptotic rate (17.6 ± 3.5% and 19.2 ± 6.7% versus 36.0 ± 3.7%, *P* < 0.05), while VEGF was 28.3 ± 3.3%; ^*∗*^ *P* < 0.05 versus control; *n* = 6 in each group. Bar scale = 80 *μ*m.

**Figure 3:**
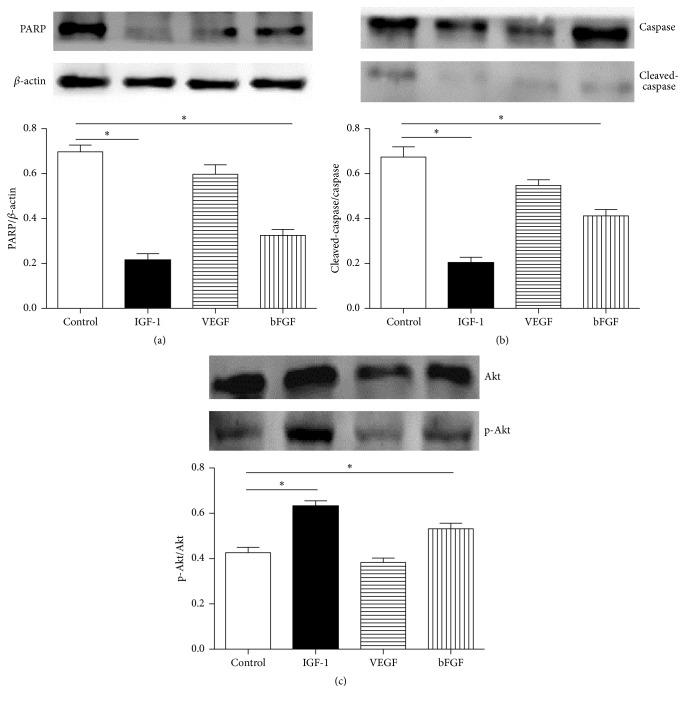
The protein abundance of p-Akt/Akt, PARP, and cleaved-caspase-3/caspase-3 in bone marrow-derived mesenchymal stromal cells (BMSCs). (a, b) The protein abundance of PARP and cleaved-caspase-3/caspase-3 was much lower following IGF-1 and bFGF preconditioning (0.21 ± 0.03 and 0.32 ± 0.01 versus 0.69 ± 0.03; 0.20 ± 0.02 and 0.41 ± 0.01 versus 0.68 ± 0.03, resp.), while the group with VEGF had the expression of PARP and cleaved-caspase-3/caspase-3 at 0.59 ± 0.06 and 0.54 ± 0.02, respectively. (c) The protein abundance of p-Akt was much higher in BMSCs plus bFGF and BMSCs plus IGF-1 than control (0.54 ± 0.03 and 0.62 ± 0.02 versus 0.41 ± 0.04, resp.), while VEGF group was 0.38 ± 0.03; ^*∗*^ *P* < 0.05 versus control; *n* = 6 in each group.

As shown in Figure 2(a), the flow cytometry assay showed that BMSCs treated with IGF-1 and bFGF underwent less apoptosis rate when exposed to hypoxia (10.2 ± 1.4% and 10.7 ± 2.5% versus 26.1 ± 3.4%; *P* < 0.05; *n* = 6 in each group) compared with control, while the apoptosis rate of VEGF was 20.7 ± 1.9%. And BMSCs with IGF-1 and bFGF showed a lower calculated apoptotic rate compared with control (17.6 ± 3.5% and 19.2 ± 6.7% versus 36.0 ± 3.7%, *P* < 0.05), while VEGF was 28.3 ± 3.3% according to TUNEL assay (Figure 2(b), *n* = 6 in each group). The protein abundance of PARP and cleaved-caspase-3/caspase-3 was much lower following IGF-1 and bFGF preconditioning compared with control (PARP: 0.21 ± 0.03 and 0.32 ± 0.01 versus 0.69 ± 0.03; cleaved-caspase-3/caspase-3: 0.20 ± 0.02 and 0.41 ± 0.01 versus 0.68 ± 0.03, *P* < 0.05, resp.), while the group with VEGF had the expression of PARP and cleaved-caspase-3/caspase-3 at 0.59 ± 0.06 and 0.54 ± 0.02 (Figures 3(a) and 3(b), *n* = 6 in each group).

### 3.3. Tracking Fluc Signal of BMSCs* In Vivo*

**Figure 4:**
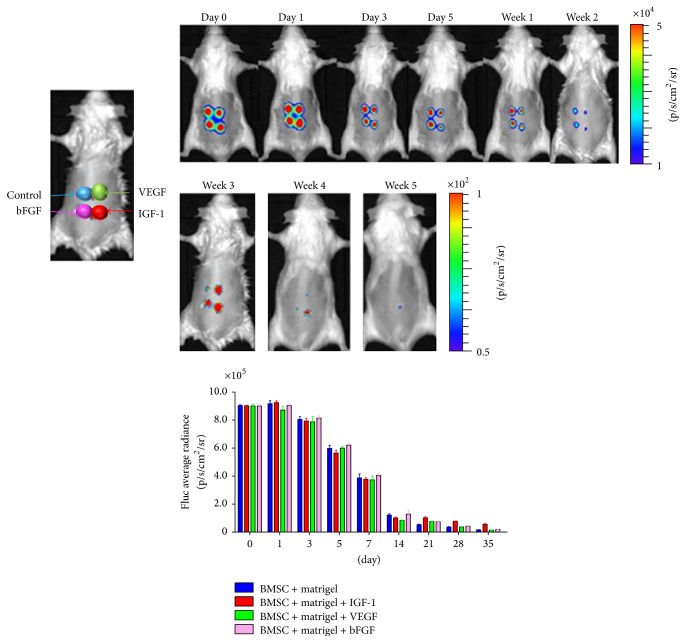
Bioluminescence imaging of the effects of VEGF, bFGF, and IGF-1 on bone marrow-derived mesenchymal stromal cells (BMSCs) survival* in vivo*. The densities of Fluc signals were similar among all groups (BMSCs + matrigel: 9.26 ± 0.22 × 10^5^ p/sec/cm^2^/sr; BMSCs + matrigel + VEGF: 8.67 ± 0.57 × 10^5^ p/sec/cm^2^/sr; BMSCs + matrigel + bFGF: 9.20 ± 0.35 × 10^5^ p/sec/cm^2^/sr; BMSCs + matrigel + IGF-1: 9.31 ± 0.57 × 10^5^ p/sec/cm^2^/sr) within 5 h. Two weeks later, the densities of Fluc signals were attenuated; five weeks later, the ratio of signals of BMSCs + matrigel + IGF-1 group to BMSCs + matrigel group (21%) was significantly higher than that of the other groups (<10%); *P* < 0.05; *n* = 6 in each group.

The densities of Fluc signals were similar among all groups (BMSCs + matrigel: 9.26 ± 0.22 × 10^5^ p/sec/cm^2^/sr; BMSCs + matrigel + VEGF: 8.67 ± 0.57 × 10^5^ p/sec/cm^2^/sr; BMSCs + matrigel + bFGF: 9.20 ± 0.35 × 10^5^ p/sec/cm^2^/sr; BMSCs + matrigel + IGF-1: 9.31 ± 0.57 × 10^5^ p/sec/cm^2^/sr) within 5 h. Two weeks later, the densities of Fluc signals were attenuated; five weeks later, the ratio of signals of BMSCs + matrigel + IGF-1 group to BMSCs + matrigel group (21%) was significantly higher than of the other groups (<10%, *P* < 0.05) (Figure 4, *n* = 6 in each group).

### 3.4. Apoptosis of BMSCs* In Vivo*

**Figure 5:**
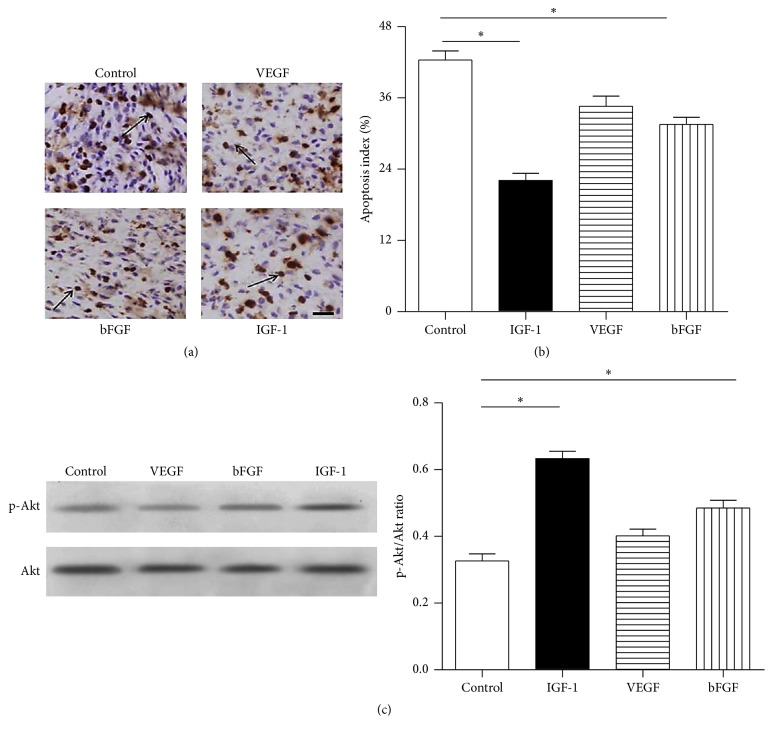
*In vivo* experiment. (a) DAB assay analysis of the efficacy of VEGF, IGF-1, and bFGF plus matrigel* in vivo*; the apoptotic nuclei shrank and turned brown. (b) Apoptosis index of BMSCs treated with VEGF, IGF-1, and bFGF, respectively, compared with control; BMSCs treated with either matrigel plus bFGF or matrigel plus IGF-1 extended BMSCs' survival (32.00 ± 1.63% and 22.66 ± 1.69% versus 42.33 ± 2.05%), while matrigel plus VEGF was 34.75 ± 3.50%; (c) p-Akt/Akt ratio of BMSCs treated with VEGF, IGF-1, and bFGF, respectively; BMSCs treated with matrigel plus bFGF and matrigel plus IGF-1 showed higher ratio of p-Akt/Akt compared with control (0.49 ± 0.03 and 0.63 ± 0.03 versus 0.33 ± 0.02), while matrigel plus VEGF group was 0.40 ± 0.02; ^*∗*^ *P* < 0.05 versus control; *n* = 6 in each group. Bar scale = 40 *μ*m.

DAB assay was performed to analyze the efficacy of VEGF, IGF-1, and bFGF plus matrigel. The apoptotic nuclei shrank and turned brown. Compared with control, BMSCs treated with either matrigel plus bFGF or matrigel plus IGF-1 extended BMSCs' survival (32.00 ± 1.63% and 22.66 ± 1.69% versus 42.33 ± 2.05%, *P* < 0.05) while matrigel plus VEGF was 34.75 ± 3.50% (Figures 5(a) and 5(b), *n* = 6 in each group).

### 3.5. Activation of p-Akt/Akt in BMSCs after Growth Factor's Administration

For* in vitro* part, the protein abundance of p-Akt was much higher in BMSCs plus bFGF and BMSCs plus IGF-1 than in control (p-Akt/Akt rate: 0.54 ± 0.03 and 0.62 ± 0.02 versus 0.41 ± 0.04, resp., *P* < 0.05), while VEGF group was 0.38 ± 0.03 (Figure 3(c), *n* = 6 in each group). For* in vivo* part, BMSCs treated with matrigel plus bFGF and matrigel plus IGF-1 showed higher ratio of p-Akt/Akt compared with control (0.49 ± 0.03 and 0.63 ± 0.03 versus 0.33 ± 0.02, *P* < 0.05), while matrigel plus VEGF group was 0.40 ± 0.02 (Figure 5(c), *n* = 6 in each group).

## 4. Discussion

Heart failure (HF) has become a public health burden worldwide, which usually results from the dysfunction of myocardium. Cellular therapy is a promising therapeutic strategy for the cardiac regeneration. Despite controversial results on this subject [30], stem cell therapy in HF has been intensely investigated and appears to be a hotspot with its promise.

Growth factors like VEGF, bFGF, and IGF-1 are thought to promote many kinds of cells' proliferation, including stem cells, which we have mentioned before. But our experiment showed a noticeable difference. In the present study, we found that, with hypoxia preconditioning for 24 h, IGF-1 and bFGF protect BMSCs from apoptosis at a higher level than VEGF by* in vitro* study, which was promising in clinical usage. As we know, myocardium infarction or ischemia always accompanied hypoxic state. Without timely treatment, myocardium could be permanently damaged. Since stem cell therapy has emerged as a promising method for treatment of ischemic heart disease [31], IGF-1 and bFGF could possibly help BMSCs function better based on our preliminary result.

The reason why growth factors functioned differently may be related to the individual features of different growth factors and different cells. Adipose-derived mesenchymal stem cells (ADSCs) are also multipotent stem cells. They originate from the vascular-stromal compartment of fat tissue and could release multiple angiogenic growth factors and cytokines/chemokines, which suggest that they may have potential as a useful cell source for therapeutic angiogenesis [32]. Our former experiment [33] also proved that ADSCs' engraftment improved blood perfusion recovery, ambulatory performance, and prognosis of the ischemic hind limb* in vivo*.

It is well known that VEGF is essential to vasculogenesis and the* de novo* formation of blood vessels from vascular precursor cells. Carmeliet et al. [34] and Ferrara [35] found that loss of a single VEGF allele in mouse models leads to gross developmental deformities in the forming vasculature and embryonic death between days 11 and 12. Also, VEGF accelerated ADSCs' proliferation [36, 37]. Because BMSCs did not have potential of angiogenesis as ADSCs [38], VEGF may not facilitate BMSCs' survival notably, which is consistent with our results in this study.

Basic fibroblast growth factor (bFGF) is a potent mitogen that stimulates the growth and differentiation of a broad spectrum of mesodermal and neuron-ectodermal cell types [39]. BMSCs have a fibroblast-like cell morphology and Moreau et al. [40] employed a two-dimension tissue culture model demonstrating that human BMSCs had the potential to be differentiated into fibroblasts. And bFGF undoubling has distinguished positive effect on fibroblasts. Besides, BMSCs get innate potential of osteoblastic differentiation based on their origin, which was also observed in our group before [41], and IGF-1 is a key factor in the endocrine regulation of body composition and enhances the differentiated function of the osteoblast [42, 43]. Therefore, these two growth factors could favor BMSCs' survival according to their close connections to BMSCs' feature and potential. Our results also showed that IGF-1 and bFGF significantly decreased the apoptosis rate of BMSCs under hypoxia environment.

Molecular imaging enables noninvasive visualization of cells' distribution and long-term viability. Among the imaging modalities, reporter genes-based bioluminescent imaging (BLI) is an ideal technique for its high throughput and low signal-to-noise ratio [44] in stem cell monitoring* in vivo*. Our group has employed this technique in monitoring stem cells' fate* in vivo* [33, 45–50]. In the present work, we chose *β*-actin-Fluc^+^ transgenic FVB mice for quantitative monitoring, which helped us in long-term tracking cells' fate and provided novel insights into intuitively identifying cells after transplantation. By molecular imaging, we found that the regions preadministered with the mixture of matrigel and IGF-1 and matrigel and bFGF had much stronger densities of signals than VEGF and control group, especially the group of matrigel plus IGF-1. These data suggested that the synergism of matrigel and IGF-1 played significantly positive roles in BMSCs engraftment and prolonging cells' viability.

Akt is a key molecule in the regulation of various signaling pathways in cell metabolism, proliferation, survival, growth, and angiogenesis [51]. In the present study, we also focused on the molecular mechanism of growth factors working on BMSCs* in vitro* or* in vivo*. We found that bFGF and IGF-1 were associated with higher p-Akt/Akt ratio (VEGF < bFGF < IGF-1) in protecting mouse BMSCs from apoptosis, which indicated that the phosphorylation of Akt contributed to this process. Therefore, we infer that bFGF and IGF-1 favored BMSCs' survival* via* p-Akt/Akt pathway.

There are still some limitations in this study; for example, the pathway of bFGF and IGF-1 functioning should be clarified in future work. Although we have got some preliminary results about optimizing stem cells' fate in heart regeneration, much more work is still under investigation.

## 5. Conclusions

Our findings suggest that bFGF and IGF-1 benefit in facilitating BMSCs' survival* in vitro* and improving transplanted cells' fate* in vivo*. Reporter gene-based BLI is valuable in tracking cells' fate after transplantation. Although further evidence is needed, this work implied that both IGF-1 and bFGF, especially IGF-1, are a promisingly strong helper in BMSCs transplantation in tissue engineering.
